# Drivers of sustainable practices in a developing country’s garment and textile industry: The role of sustainability challenges, limited material access, and economic constraints

**DOI:** 10.1371/journal.pone.0338270

**Published:** 2026-05-29

**Authors:** Kweku Safo-Ankama, Lord Emmanuel Yamoah, Naa Omai Sawyerr, John Amoah

**Affiliations:** 1 Department of Textile Design and Technology, Takoradi Technical University, Takoradi, Ghana; 2 Department of Procurement and Supply, Takoradi Technical University, Takoradi, Ghana; 3 Department of Marketing and Entrepreneurial Studies, Takoradi Technical University, Takoradi, Ghana; Indira Gandhi National Tribal University, INDIA

## Abstract

Sustainable practices in small and medium-sized enterprises (SMEs) are critical for environmental resilience and economic viability, particularly in developing economies like Ghana’s garment and textile sector. This study investigates the drivers of sustainable practices among SME drivers in the Western Region of Ghana, focusing on sustainability challenges, limited access to materials, and economic constraints. Employing a quantitative approach, data were gathered from textile and garment SMEs via simple random sampling through an online and offline approach. Partial Least Square Structural Equation Modelling (PLS-SEM 4.0) was used in processing and analysing the 315 valid responses gathered. Findings revealed that sustainability challenges and limited material access have a significant effect, while economic constraints have an insignificant effect. These results underscore the need for targeted interventions, including investments in recycling technology and stakeholder collaboration. The study contributes to SME sustainability literature in developing contexts and offers practical recommendations to foster eco-friendly production, job creation, and regional leadership in West African textile manufacturing.

## 1. Introduction

Sustainable practice is seen as a pivotal strategy to achieve firms’ operational efficiency and gain a competitive advantage in a recent saturated market [1]. Different economic sectors, including the textile industry, have considerable social, economic, and environmental challenges, despite their enormous contributions to developing countries’ economies [2], in terms of employment and gross domestic product generation. The textile sector, according to [3], significantly contributes to environmental contamination at every stage of its supply chain due to its high levels of waste generation, air pollution, water depletion, carbon emissions, and greenhouse gas emissions [4]. In order to lessen the industry’s environmental impact, it is crucial to incorporate comprehensive sustainability measures across textile industry operations [5,6].

Ghana’s garment and textile industry stands at a critical juncture, confronting entrenched sustainability challenges alongside opportunities for transformative change. As a historically vital manufacturing sector and a vibrant repository of cultural heritage, embodied in iconic Kente and Adinkra textiles, it faces multifaceted pressures shaping its sustainability trajectory [7]. Key environmental concerns include wastewater pollution from dyeing processes, voluminous textile waste, and overexploitation of natural resources such as water and land, all underscoring the urgent need for mitigation strategies [8]. These issues are exacerbated by socioeconomic and structural barriers, including the dominance of imported second-hand clothing, limited access to eco-friendly raw materials and technologies, and inadequate regulatory frameworks that hinder effective sustainability governance.

In particular, material shortages and the high costs of green materials compel companies in Ghana’s garment and textile sector to innovate with resource-saving approaches, turning constraints into sustainability drivers. However, as [5] argues, these factors often act as barriers rather than facilitators, due to economic hurdles like elevated production costs, limited funding, and intense competition from cheap imports.

Despite these challenges, the interconnected issues of sustainability, material shortages, and economic constraints are driving greater awareness and the adoption of sustainable practices. This shift is fueled by urgent environmental needs, cultural heritage priorities, and emerging policies promoting circular economy models and green innovation. Similarly, [9] highlights that Ghana’s garment and textile industry, a vital economic and cultural sector, faces severe sustainability threats that undermine both ecological balance and business viability. These problems are intensified by limited access to eco-friendly materials, unaffordable green technologies, scarce production resources, fierce competition from imported second-hand clothing, and burdensome regulations. While economic barriers hinder firms’ investments in sustainable innovations, material scarcity paradoxically spurs creative alternatives, turning constraints into catalysts for change. Yet, as [10,11] emphasise, major knowledge gaps remain about how these interconnected challenges affect motivation and adoption of sustainable practices in Ghana’s garment and textile sector.

Policymakers, industry stakeholders, and researchers must understand how these factors drive the adoption of sustainable practices in Ghana’s garment and textile industry. This awareness can help the sector achieve environmental responsibility alongside economic viability. The primary aim of this research is to examine the key determinants of sustainable practices in Ghana’s garment and textile sector. The findings will inform more effective strategies to promote sustainability while preserving the industry’s vital cultural and economic roles.

## 2. Theoretical foundation of the study

The Sustainable Development (SDT) theory offers a permanent paradigm in terms of which the development of environmental, economic, and social priorities could be promoted simultaneously in the garment and textile sector, particularly in developing economies such as Ghana. The Brundtland Commission (1987) was the first to introduce SDT, and it defined sustainability as the ability to meet the present needs without harming the needs of future generations. This theory has been pegged in the triple bottom line model, which focuses on the three interrelated propositions, environmental integrity, economic resilience, and social equity [12]. The new empirical studies available on the subject justify the significance of SDT in coming up with sustainable practices within the local industries. This support is attributed to the fact that the companies which are involved with the principles of sustainable development are more successful in terms of competitiveness, their environmental performance, and the trust of their stakeholders [13,14]. Besides, the theory accepts context-specified applications that can be provided with corresponding adaptations to meet small-scale producer-specific cultural, economic, and technological settings [15].

The SDT was used within the framework of this research to understand sustainability holistically as compared to environmental compliance. The implementation of the given theory will aid in finding the proactive strategies, including material supply and use, manufacturing operations, employment relations, and so on, within the supply chain pattern. Regarding the contemporary issues of this industry that involve the lack of legal regulation and the unreliability of technology, the SDT can be applied to design an orderly method of determining the alternatives, such as the utilisation of native fibre, ecologically sound procedures of dyeing, and closed-loop production systems [14]. This association by SDT enables the placement of the concept of sustainability not only as the normative goal but also as a feasible method of inclusive development. It presents a possibility to explore the interaction between the structural challenges and the factors of innovation, collaboration, and consumer demand boosting [16]. In addition, the theory adapts to the new technologies that will enhance the traceability, resources and visibility of the value chain optimisation. In this perspective, SDT can be viewed as a diagnostic and prescriptive instrument as it provides a holistic approach to reconsidering the literature on the garment and textile industry in a manner that respects ecological equilibrium, encourages economic inclusion and preserves cultural heritage.

## 3. Literature review and hypotheses development

### 3.1. Drivers of sustainable practices

Sustainability practice within small enterprises in the garment and textile industry in Ghana is not just a matter of aspiration but also founded on the ability of enterprises to move across systemic constraints. The motivators of sustainable practices in this case happen to be a reaction to a group of interrelated obstacles that are either institutional, material or financial. The invention of such innovations as the use of vegetal dyes or the use of textile waste is typically created in times of shortage [17]. Alternatives, including the community-based interventions, such as cooperatives and resource-sharing programs, were also regarded as a possible way out of the current constraints in the economy and the infrastructure [13–18]. Furthermore, the information about cultural issues, historical approaches, and informal learning strategies tends to play a central role in sustaining the environmentally responsible local and contextually embedded behaviours [16]. Thus, the facilitators of the sustainable practices are not unilateral antecedents but are relationally constructed through the dynamic-boundary negotiation of the constraints that foreshadow hybrid models of sustainability based on the grassroots innovation and socio-economic resiliency.

### 3.2. Challenges to green sustainability

The concept of sustainability being implemented within the garment and textile sector, which, in this particular case, involves small-sized manufacturing units in developing economies, faces a mountainous load at the systemic level. These include bad regulatory governance, bad coordination of the institutions, bad waste management and lack of proper sustainability policy on the industry [19,20]. The practice with small-scale producers in Ghana is usually informal and does not feature in national agendas, resulting in peripheral green practices. Local artisans’ networks, local cooperatives and NGOs are developing locally owned recycling systems with new forms of circular production [21]. This has consequences of proving that despite the imposition of constraints by system-level issues, it can also encourage local sustainability changes. They describe such issues that are shaping the design and development of sustainability drivers according to [15], contingent upon the collaboration and exchange of information. We have hypothesised, based on this discussion, that;

*H1: Sustainability challenges have a significant relationship with drivers of green sustainable practices*.

### 3.3. Limited green sustainability materials access

The sustainable materials are significant in terms of green practices undertaken by the garment and textile industry, as they are conditional on the use of more environmentally friendly materials like organic cotton, biodegradable dyes, etc. In less developed economies, these materials cannot be easily obtained due to the absence of agricultural investment, poor infrastructure to process them, and fragmented markets [22,23]. This makes them employ artificial or foreign raw materials, thereby lowering the sustainability. Similar to big data, which requires advanced structures, small amounts of green materials require combined interventions to be achievable. A study by [24] discovered that responsible sourcing and eco-labels are better due to the existence of green materials. The access is not only low, but it also weakens the technical feasibility, and it also diminishes the impact of other sustainability drivers like consumer demand. The conflict of material constraints and economics, technology, and policy rates affects the value chain structure and scalability of the sustainable practices [25]. Therefore, the following hypothesis is used in the study;

*H2: Limited access to green sustainability materials has a significant relationship with drivers of sustainable practices*.

### 3.4. Economic constraints

Economic constraints play a central but often ambivalent role in shaping sustainable practices within developing countries’ garment and textile industries. Low profit margins, limited access to capital, and high relative costs of sustainable inputs and technologies act as key barriers to the adoption of green manufacturing. Economic constraints serve as an important factor in achieving sustainability in the small-scale garment and textile sector in Ghana, since it implies that the industry is constrained by financial concerns that influence the choice of materials, labour, technology, and cost. The initial costs, which may be prohibitive when it comes to small enterprises, may include the implementation of sustainable practices through the purchasing of eco-materials, cleaner technologies, or certifications [26,27]. Similar to the case of big data analytics, the need to become sustainable should be complemented by the development of particular financial policies and strategies [28]. Otherwise, sustainability initiatives will be slowed down without such support. However, the recent studies suggest that those economic limitations have already led to some adaptive measures, including innovation hubs that are donor-based and collaborative funding projects that enable the possibility of sustainable experimentation despite the financial limitations of the environment [13]. In addition, economic constraints can either compound or lessen the performance of other sustainability concerns, and this proves the plausibility of financial competence in the sustainability continuum. Since it was in line with the SDT, in this study, the hypothesis was:

*H3: Economic constraints have a significant relationship with the drivers of sustainable practices.*

**Fig 1:**
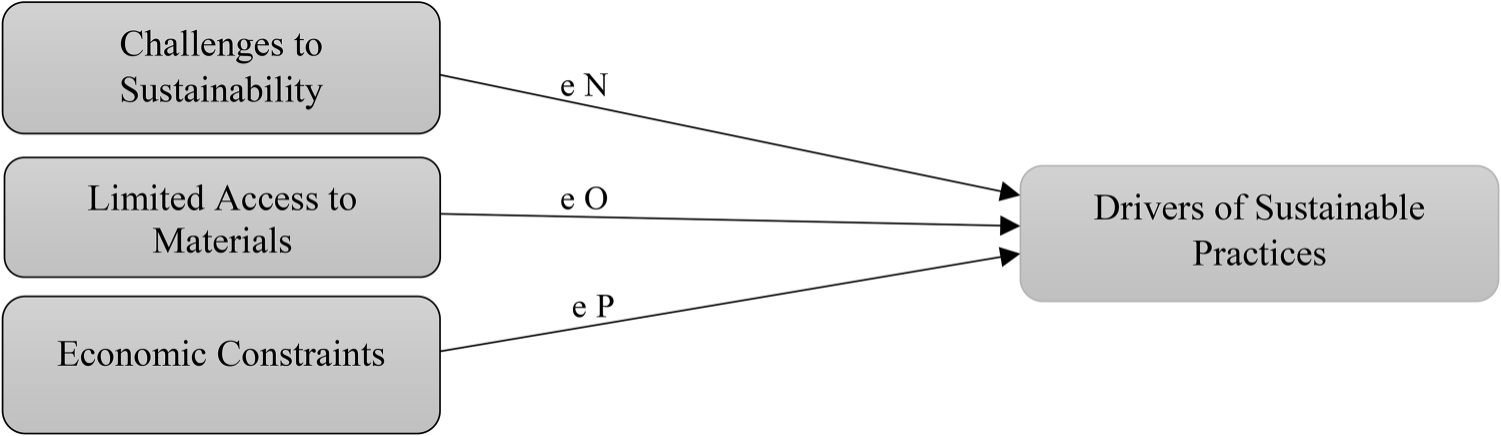
Proposed conceptual framework.

Based on the reviewed literature, the study therefore proposed the following conceptual framework, as seen in Fig 1 below.

## 4. Materials and methods

### 4.1. Data collection and sampling

Quantitative approach was deployed in the study. The use of the quantitative approach was to minimise the researchers’ bias through standardised tools like surveys and statistical analysis, ensuring consistent, replicable results across large samples. The sample was drawn from a list of registered Garment and Textiles SMEs in the manufacturing and production sectors from the National Board for Small-Scale Industries (NBSSI) regional office, Western region. SMEs in the manufacturing and production sectors within five districts were used for the data collection, specifically Essikado-Ketan, Takoradi, Efia, Kwesimitsim and Sekondi. The selected districts are notable for their business operations within the region. Respondents were drawn using simple random sampling from the accessible population, collected via online and offline questionnaires, to ensure representation across the targeted districts [29–32]. Both online and offline data collection approaches were deployed. A cross-sectional research design was used in the study since the data were collected once for its analysis. To be more specific, 398 structured questionnaires were administered through both online and offline means. Out of this, 350 responses were received, with a non-sampling error of 35 questionnaires. This affirmed that the 35 non-sampling error questionnaires indicated incomplete questionnaires among other factors, which makes it invaluable for it to be used for the final data processing and analysis. Given this, a total of 315 valid responses were used in the data processing and analysis. A five-point Likert scale, particularly (1 = strongly disagree, 5 = strongly agree) was adopted. The structured questionnaire was answered by owner-managers of the selected SMEs in the manufacturing and production sector. Participants used in the study’s data collection were assured of confidentiality and also promised that the data gathered was for academic purposes. Given this, participants’ informed consent was sought. A total of 45 respondents were used in the pilot study process to ensure corrections and modifications of the study’s research questions. Four months were used in gathering the needed data from January to April 2025. In addition to that, the participants were assured of anonymity, which served to motivate them to provide sincere feedback and to protect their identity. The study applied the partial least square structural equation modelling (PLS-SEM) 4.0 version, which is suitable when complex predictive equations are used [33]. This allowed latent variable tests and path connections, bearing in mind the non-normality of organisational research data. Table 1 below depicts the details of the respondents’ profiles.

**Table 1:** Respondents’ Profile.

Details	Groups	Frequency (N = 315)	Percentage
Age	Below 30	96	30.47
	31-40	154	48.89
	41-50	38	12.06
	51-60	27	8.57
Gender	Male	194	61.59
	Female	121	38.41
Work Exp.	1-3	68	21.59
	4-7	102	32.38
	8-15	103	32.70
	Above 16	42	13.33
Location of your business	Efia	74	23.49
	Essikado-Ketan	68	21.59
	Takoradi	121	38.41
	Sekondi	36	11.43
	Kwesimitsim	16	5.08
Academic Qualifications	Senior High School Cert.	15	4.76
	Diploma/HND	78	24.76
	Graduate	147	46.67
	Postgraduate	75	23.81

Data retrieved from online and field collection: January-April, 2025.

### 4.2. Measurement and instrument development

All the items or scales used in the questionnaire were adapted and modified from existing studies, such as Challenges to Sustainability [34–36], Limited Access to Materials [37,38], Economic constraints [39,40], and Drivers of Sustainable Practices [41,42]. The questionnaire design followed best practices to minimise common method bias in measurement error, as indicated by [43]. This was achieved through clear wording, logical question ordering, and optimised item sequencing to ensure high data quality and respondent understanding. To further mitigate potential bias, questions measuring independent and dependent variables were distributed across different survey sections. This procedural remedy, supported by prior research, reduces artifactual covariance [44]. The questionnaire underwent pilot testing and expert review to evaluate its reliability and validity. It was subsequently revised based on the feedback received. The reliability of the construct was measured using Cronbach alpha for internal consistency.

### 4.3. Common method bias

To address potential common method bias (CMB), the researchers implemented both procedural and statistical controls. Following [45], the study used clear construct items, assured participant confidentiality and anonymity on the cover page, and informed respondents of their voluntary participation and right to withdraw at any time. Additionally, we conducted multicollinearity analysis using variance inflation factor (VIF) values to assess common method variance (CMV). No VIF values exceeded the critical threshold of 10, and all constructs surpassed the recommended minimum of 0.5, as per [46,47]. These findings confirm that multicollinearity and CMV do not pose significant concerns. Table 2 reports the minimum CMB observed.

**Table 2:** Construct, Indicator, Loading, VIF, CR, AVE, and CA.

Constructs	Indicator	Factor Loading	VIF	CA	CR	AVE
Challenges to Sustainable Practices	1. It is difficult for my business to adopt sustainable garment and textile sourcing practices due to the high cost of eco-friendly materials	0.794	1.573	0.793	0.795	0.617
	2. My business lacks sufficient knowledge and information about sustainable and eco-friendly garment and textile sourcing options.	0.763	1.550			
	3. The complexity of sourcing and verifying sustainable garments and textiles makes it challenging to implement green practices.	0.794	1.658			
	4. There is insufficient consumer demand for sustainably sourced garment and textile products in my market.	0.790	1.614			
Limited Access to Materials	1. My business faces difficulties sourcing enough locally produced cotton or eco-friendly raw materials for garment and textile production.	0.750	1.486	0.767	0.769	0.589
	**2.** The high cost of importing sustainable garment and textile materials limits my ability to adopt green practices in my business.	0.746	1.421			
	**3.** Delays and complications in the import process make it challenging to obtain the materials needed for eco-friendly garment and textile production.	0.770	1.520			
	**4.** There is a lack of reliable local suppliers for sustainable or eco-friendly garment and textile materials.	0.801	1.597			
Economics Constraints	1. Limited access to affordable financing makes it difficult for my business to invest in sustainable garment and textile sourcing.	0.819	1.360	0.739	0.805	0.582
	**2.** High production costs prevent my business from adopting green and eco-friendly garment and textile practices.	0.643	1.149			
	**3.** Government support and incentives for sustainable garment and textile sourcing are insufficient for small-scale businesses like mine.	0.814	1.378			
Drivers of Sustainable Practices	**1.** Government policies and incentives encourage my business to adopt green and eco-friendly garment and textile sourcing practices.	0.774	0.165	0.830	0.831	0.595
	2. Access to training and information about sustainable materials motivates my business to implement environmentally friendly sourcing methods.	0.792	1.667			
	3. Rising consumer demand for eco-friendly products influences my business to prioritise sustainable garment and textile sourcing.	0.761	1.767			
	**4.** Collaboration with local and international organisations supports my business in adopting sustainable garment and textile practices.	0.786	1.621			
	**5.** The availability of locally sourced natural fibres (such as cotton, kenaf, or jute) makes it easier for my business to pursue sustainable garment and textile production.	0.741	1.762			

## 5. Data analysis and results

### 5.1. Assessment of the measurement model

Partial Least Square Structural Equation Modelling (PLS-SEM) was used to examine relationships among latent constructs. The measurement model was evaluated in several steps, including factor loadings, convergent validity, and discriminant validity. First, indicator factor loadings were inspected to ensure they met the minimum threshold of 0.5, retaining only those that improved overall model fit [48,49]. Next, internal consistency was assessed using Cronbach alpha and composite reliability (CR), while convergent validity was confirmed by average variance extracted (AVE), following [48]. All constructs exceeded the acceptable internal consistency threshold of 0.7 and demonstrated strong convergent validity. Table 2 summarises key measurement items, including factor loadings, Cronbach alpha, CR, AVE, and VIF values for all constructs.

Discriminant validity was established using the Fornell-Larcker criterion, which requires the square root of each construct’s AVE to exceed its correlations with other constructs. Results showed all constructs had AVE values above the recommended 0.5 threshold, with inter-construct correlations lower than their respective AVE square roots. This confirms the constructs.

### 5.2. Assessment of the structural model and hypothesis testing

The third step in the structural model analysis involved hypothesis testing and R^2^ calculation, following [50]. Before this, collinearity among predictor variables was assessed using variance inflation factor (VIF) values, which ranged from 1.3 to 1.7, within acceptable limits, confirming effective model performance.

**Fig 2:**
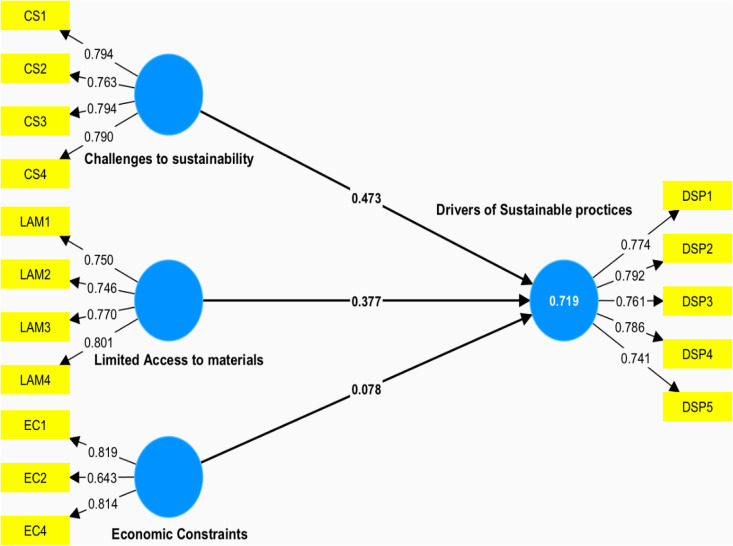
Estimated model.

To address potential common method bias (CMB) inherent in cross-sectional survey data, preventive measures were implemented per Scott and Bruce (1994). Harman’s one-factor test revealed three eigenvalues exceeding 1, accounting for 70% of total variance, with no single factor dominating. Recognising Harman’s test limitations, we additionally applied the Partial General Factor approach within the PLS model, as recommended by [51]. Once measurement model reliability and validity were confirmed, explanatory power was evaluated via R^2^ values (Table 3, Fig 2). Hypothesis testing results, as seen in Table 4, indicated that two of the hypotheses were insignificant, while one was not.

**Table 3:** Discriminant Validity.

Constructs	Challenges to sustainability	Drivers of Sustainable Practices	Economic Constraints	Limited Access to materials
Challenges to sustainability	**0.785**			
Drivers of Sustainable Practices	0.798	**0.771**		
Economic Constraints	0.668	0.649	**0.763**	
Limited Access to materials	0.725	0.772	0.678	**0.767**

**Table 4:** Hypothesis Testing.

Construct	Original Sample	Sample Mean	Standard Deviation	T Statistics	P values	Decision
H1: Challenges to Sustainability → -Drivers of Sustainable Practices	0.473	0.474	0.059	7.999	0.000	**Accepted**
H2: Economic Constraints → Drivers of Sustainable Practices	0.078	0.079	0.052	1.491	0.136	**Not Accepted**
H3: Limited Access to Materials → Drivers of Sustainable Practices	0.377	0.376	0.055	6.879	0.000	**Accepted**

## 6. Discussion

The study was set to investigate Drivers of Sustainable Practices in a Developing Country’s Garment and Textile Industry with the Role of Sustainability Challenges, Limited Material Access, and Economic Constraints**.** Based on the results, it can be concluded that sustainability-related problems (H1) and the lack of materials (H3) are significant factors affecting sustainable behaviour in Ghana’s garment and textile industry, while economic factors (H2) are not statistically significant. It confirms the fact that sustainability in developing countries’ textiles is determined by structural and contextual issues, but not solely by financial capability. In support of this, for example, there have been various studies conducted regarding Ghana’s textile industry, indicating the ways sustainability can be developed through structural and contextual issues like environmental damage, legal issues, and market pressure [52].

Relative to (H1), it can be inferred that challenges to sustainability influence sustainable practices consistent with [52]. This implies that organisations that comply with pressure factors from the environmental, regulatory, and other stakeholders can bring about changes in their sustainable practices. According to the literature, environmental damage, pollution, and lack of mechanisms result in situations where firms must engage in sustainable practice to solve problems [52,53]. The results conform to existing studies that the institutional pressure of regulatory pressure, customer pressure, and competition drives sustainability practice in the textile industry, especially in emerging countries [54,55]. Thus, the increased interactions of businesses with the global market environment compel them to adopt sustainable practices to legitimise themselves and remain competitive.

However, H2 was not supported, meaning economic constraints do not promote sustainable practices in the textile industry. Contrary to the empirical findings that sustainable practices lead to cost savings and operational benefits over time [3]. This implies economic constraints are practically a core dimension of sustainability. Although cost is an essential aspect, research reveals that companies in emerging markets engaged in textiles tend to be influenced more by factors other than cost, such as institutional factors, stakeholder demands, and market access [56,57]. Perhaps, the negative effect is an indication that the Ghanaian textile industry sees economic factors as consequences of achieving sustainable business operation and not vice versa. In most developing economies, although there are certain financial restrictions, companies continue to be subjected to external influences such as environmental protection laws and their involvement in global supply chains. These external influences could account for the absence of economic limitation as a predictor in this study.

Similarly, the validation of H3 means that material shortages can serve as a source for sustainability innovation. The experience from Ghana’s textile industry demonstrates that a lack of sustainable materials and technologies limits manufacturing activities while, at the same time, fostering innovative solutions like recycling and efficiency [52]. Such results align with studies on the sustainability transition within the textile industry, emphasising the significance of innovations related to technologies and processes that contribute to environmental sustainability under resource shortages [13–18]. Thus, the scarcity of raw materials in manufacturing industries, coupled with increased waste, significantly contributes to the growing interest in the circular economy. Businesses in emerging markets address their shortages through effective utilisation of materials and new manufacturing techniques, indicating that scarcity might inspire innovation instead of hindering it. [4] affirm that scarcity of resources presents an opportunity for firms to project their positive sustainability practices, such as retrofitting, safer work environment, energy and resource saving initiatives.

## 7. Implications

### 7.1. Theoretical implications

This study advances understanding of sustainable practices in Ghana’s garment and textile industry by highlighting how contextual challenges influence adoption. It bridges gaps in sustainability literature specific to developing economies. First, the study reinforces and extends theories and concepts of sustainability, particularly research on the manufacturing industry. The findings demonstrate that sustainability functions not merely as a voluntary strategic tool but as a necessary response to institutional, environmental, and operational threats. This supports the core tenet of Sustainable Development (SDT) theory that organisational behaviour is shaped by external pressures such as regulations and norms [58,59]. Theoretically, this implies that for firms in emerging markets, pursuing sustainability is a legitimacy-seeking mechanism aimed at aligning operations with international standards, thereby extending the theory’s applicability to contexts where global supply chain pressures intersect with local institutional voids.

Second, the study advances resource-based by introducing a scarcity-driven innovation mechanism. While organisational resources traditionally emphasise how unique and valuable resources shape competitive advantage [60], this study shows that resource scarcity itself can act as a catalyst for sustainable innovation. The findings imply that limited material access does not simply constrain firms; it can force creative problem-solving and eco-innovation [61]. The theoretical implication is significant for developing economies where scarcity becomes an endogenous driver of sustainability rather than merely an exogenous barrier. This reframes resource-based assumptions to account for how resource constraints, when institutional pressures are present, generate novel sustainable practices.

Third, the study challenges and refines conventional economic determinism. Contrary to the prevailing assumption that financial costs are the primary barrier to sustainability adoption [62], this study finds that firms may pursue sustainable initiatives despite economic disadvantages. This theoretical implication is twofold: (a) non-financial drivers such as supply chain dynamics, regulatory pressure, and reputational concerns can override economic constraints [63]; and (b) existing sustainability frameworks, largely developed in high-income contexts, overemphasise financial logic and underemphasise socio-institutional pressures.

### 7.2. Practical implications

The conclusions drawn from this study have implications for the various actors in the garment and textile industry. Industry actors can learn from the study that sustainability programs can be easily put in place even in situations of resource scarcity. Companies are advised to analyse the constraints that face them as opportunities to come up with innovations that can help sustainably address the issue. Scarcity of resources is not just viewed as a constraint but can also lead companies to come up with innovative ways of doing things. This is because the use of recycling and the reuse of products has been associated with sustainable production programs [64]. In addition, companies will increase their competitiveness in the international market due to increased sustainability compliance requirements in such markets.

From the policy standpoint, the research indicates that any effort aimed at promoting sustainability needs to transcend beyond the provision of financial instruments. Although finances are essential, more attention should be devoted to increasing access to sustainable material resources and providing the required infrastructures for ensuring sustainable production. The key role here can be played by policymakers who need to facilitate efficient logistics channels and promote the production of environmentally friendly materials domestically while minimising obstacles related to importing sustainable materials. This corresponds to international policy advice, which highlights the role of systemic approaches to developing sustainable logistics and locally sustainable industries [65,66]. Additionally, innovation-related policies can substantially boost the process of transformation towards sustainability.

For development agencies and other assisting entities, the results of the analysis suggest the necessity to implement capacity-building programs that would emphasise knowledge building and resource access issues. Collaboration efforts between domestic companies and foreign organisations can help reduce the current gaps and increase the use of sustainable practices within the industry, which is discussed in sustainable development models [67]. These collaborations can also help to gain new skills and technologies essential for achieving sustainability. Finally, the results obtained suggest that the shift in perception towards sustainability is crucial. Instead of treating it as an additional cost to sustain, it should be treated as a source of competitive advantage and a means to achieve better results in the future. This point of view is also supported by previous evidence indicating that sustainability can generate shared value for businesses and improve performance outcomes [68]. Hence, managers should start considering sustainability as an integral part of their business activities.

## 8. Conclusion and limitations

The study investigated the key determinants of sustainable practices in Ghana’s garment and textile sector catering on challenges to sustainability, limited access to materials, economics constriants and drivers of sustainable practices. The findings reinform more effective strategies to promote sustainability while preserving the industry’s vital cultural and economic roles.

Two out of the three hypotheses formulated were significant while one was insignificant. These findings reconcile the role of institutional settings and resource ecologies in the definition of the sustainability results in the informal sectors. The results confirm the discussion that the concept of sustainability in the Global South cannot be recognised in the same manner as it can be in instances of industrialised economies. Instead, it must be seen as a process that is affected by its constraint navigation, recombinance of resources and more of the locally based innovation. The paper assumes the role of expanding the scope of the Sustainable Development Theory.

The specified research has drawbacks despite its contributions. The study also had some limitations in the sampling in that it was restricted to the garment and textile production sector in Ghana, and thus, the study findings would not be generalised to other sectors. The use only cross-sectional design also pose as a limitation to the study findings. Furthermore, one more problem that introduces a risk of bias is the use of self-reported data, which is validated by the use of the survey instrument. Also, further studies can reintroduce mediation and moderation variables as well control variables since the present study only concentrated on the direct relationship.. Furthermore, the generalisation of the findings to West Africa countries should be done with caution since the paranetrs are different.. A longitudinal study can also be utilised to give information about the change in sustainability practices in various stages of the economy or the policy. Lastly, it will be prudent for future to consider the use of qualitative approach to obtain more insight about behavioural and cultural influences underlying sustainability adoption in informal economies.

**Consent to participate**

To agree to take part, a written statement was given.

**Publication consent**

No data of a person (individual information, photos or recordings) is used in the manuscript.

Clinical trial number: NA

Declaration Section

The Takoradi Technical University (TTU) Research Ethics Committee did not demand ethical review and approval of this proposed research since they did not demand any sensitive information or identify any participants of this research. All the processes involved in the data collection exercise were carried out to the TTU ethical requirements, that is, with confidence in all the stages involved. They had signed informed consent whereby they were aware that the survey was anonymous, and they were also made aware that the survey was purely voluntary.

## Supporting information

S1 Data Data Ankamah. (CSV)
